# The Genetic Status of the Critically Endangered Hainan Gibbon (*Nomascus hainanus*): A Species Moving Toward Extinction

**DOI:** 10.3389/fgene.2020.608633

**Published:** 2020-12-04

**Authors:** Yanqing Guo, Jiang Chang, Ling Han, Tao Liu, Gang Li, Paul A. Garber, Ning Xiao, Jiang Zhou

**Affiliations:** ^1^School of Karst Science, Guizhou Normal University, Guiyang, China; ^2^College of Life Sciences, Northwest University, Xi’an, China; ^3^State Key Laboratory of Environment Criteria and Risk Assessment, Chinese Research Academy Environmental Sciences, Beijing, China; ^4^Department of Anthropology, Program in Ecology, Evolution, and Conservation Biology, The University of Illinois at Chicago, Urbana, IL, United States; ^5^Guiyang Nursing Vacational College, Guiyang, China

**Keywords:** critically endangered, genetic status, population size, Hainan gibbon, conservation

## Abstract

The Hainan gibbon (*Nomascus hainanus*), once widespread across Hainan, China, is now found only in the Bawangling National Nature Reserve. With a remaining population size of 33 individuals, it is the world’s rarest primate. Habitat loss and fragmentation are the primary drivers of Hainan gibbon population decline. In this study, we integrated data based on field investigations and genotype analyses of 10 microsatellite loci (from fecal samples) to assess genetic diversity in this Critically Endangered primate species. We found that the genetic diversity of the Hainan gibbon is extremely low, with 7 of 8 microsatellite loci exhibiting decreased diversity. Additional molecular analyses are consistent with field observations indicating that individuals in groups A, B, and C are closely related, the female–male sex ratios of the offspring deviates significantly from 1:1, and the world’s remaining Hainan gibbon population is expected to experience continued high levels of inbreeding in the future. Given extensive habitat loss (99.9% of its natural range has been deforested) and fragmentation, this rarest ape species faces impending extinction unless corrective measures are implemented immediately.

## Introduction

Species classified by the IUCN as Endangered (EN) or Critically Endangered (CR) face an impending extinction crisis and require immediate protection. In the case of nonhuman primates, which represent the 3rd most speciose mammalian order (some 512 species, Estrada et al., 2017), 17% of species are currently listed as CR and 28% as EN (Iucn, 2020). In the case of gibbons and siamangs (Hylobatidae, Primates) or small bodied Asian apes (genera *Hylobates*, *Hoolock*, *Nomascus*, and *Sympalangus*), the threat of extinction is extremely severe. Ninety-five percent (18 of 19 species) of gibbon and siamang species are EN or CR. This includes the world’s most threatened primate species, the Hainan gibbon (*Nomascus hainanus*). Only 33 Hainan gibbons remain in the wild. In the current study, we examine the genetic status of the critically endangered primate species.

There are several evaluation criteria used to assess a species viability and conservation status for it to be assessed as CR. These include the remaining population is small, declining, and geographically restricted; the species geographic range is highly fragmentated and decreasing; and quantitative assessments indicate that the extinction risk is high (Iucn, 2004). Given the difficulty of obtaining DNA samples of highly threatened and rare primate species, population genetics have seldom been used to assess their population viability. However, population genetic assessments are essential for effective programs of species management and conservation. For example, in 1958 China established the Xishuangbanna National Nature Reserve in Yunnan Province in an attempt to protect the Critically Endangered northern white-cheeked gibbon (*Nomascus leucogenys*). In 1980, China established the Nangunhe National Nature Reserve, also in Yunnan Province, to protect the Endangered lar gibbon (*Hylobates lar*). Although both gibbon species were censused and demographic information collected, studies by Fan et al. (2013) indicate that both gibbon species have been extirpated from China. Thus, although the creation of protected areas and periodic population re-censusing are important components of a species survivorship plan, population genetics research that includes estimates of genetic diversity, effective population size, inbreeding potential, and strategies for increasing gene flow also are essential.

Many species are facing a significant threat due to anthropogenically induced habitat fragmentation and habitat loss that has resulted in previously continuous populations becoming isolated (David and Richard, 2003; Frankham, 2005; Wei et al., 2012; Estrada et al., 2017; Li et al., 2018). As subpopulations decrease in size, they also are expected to decline in genetic diversity. In general, populations with higher genetic diversity have a greater ability to adapt and respond to changing environmental conditions (Lindsey et al., 2013) and are more disease resistant (Siddle et al., 2007), then populations characterized by limited genetic diversity (Swinnerton et al., 2004; Hemmings et al., 2012). At the same time, small populations are at greater risk for inbreeding, which will increase the degree of genetic drift and allelic loss, leading to reduced genetic diversity (Frankham et al., 2009; Frankham et al., 2014).

Research in conservation genetics has found that many small and isolated populations exhibit low genetic diversity. For example, the population of northern elephant seals (*Mirounga angustirostris*) inhabiting Guadalupe Island was reduced to a relic population of only 10–30 individuals in the 19th century. By 1991, the population had increased to 127,000. Despite this rapid population increase, genetic research indicates that the genetic variation of northern elephant seals remains extremely low and has been severely affected by inbreeding and genetic drift. The genetic diversity of the population is only 45% of the original population (Hoelzel, 1997). Similarly, recent habitat fragmentation has resulted in several primate species in China (e.g., *Rhinopithecus roxellana*, *Rhinopithecus bieti*, *Rhinopithecus brelichi*, *Trachypithecus francoisi*, and *Trachypithecus leucocephalus*) distributed into small isolated subpopulations. Genetic differentiation across subpopulations is high (0.109–0.177), and in at least three of these five species, genetic drift has occurred (Liu et al., 2015). In the black snub-nosed monkey (*Rhinopithecus strykeri*), which has a remaining population of approximately 400 individuals (200 individuals in China and 200 individuals in Myanmar; Yang et al., 2019), genetic diversity in the mitochondrial control region indicates no variability (Andie et al., 2016). A greater understanding of the genetic diversity of threatened species is critical for developing effective management and conservation plans.

The Hainan gibbon (*Nomascus hainanus*) (Thomas, 1892) is endemic to China, and although it was once widespread across Hainan Province, it is now considered the world’s rarest ape, with a remaining population of only 33 individuals. These 33 Hainan gibbons are distributed in five groups (A, B, C, D, and E) that inhabit the Bawangling National Nature Reserve, which is a protected area of some 16 km^2^ (Deng et al., 2015). In the past 70 years, the Hainan gibbon population has declined by 99.4%and its habitat has declined by 99.9% (Liu et al., 1989; Zhou et al., 2005; Deng et al., 2015). Systematic field observations have been conducted in the reserve since 2001, and over the past 17 years, only two new breeding groups have been established. The genetic relationships among breeding individuals as well the sex of the offspring recently born into each family group remain unknown.

Previous genetic studies of the Hainan gibbon have focused either on broad phylogenetic analyses (Su et al., 1995; Zhang, 1995; Thinh et al., 2010a,b), or attempts to identify microsatellite diversity of the mitochondrial DNA (mtDNA) control region (Li et al., 2010; Bryant et al., 2016b). Unfortunately, sampling limitations have made it difficult to conduct a comprehensive genetic analysis, and therefore our understanding of genetic variation in this last remaining Hainan gibbon population is extremely limited.

In the current study, over the course of one year, we collected fresh fecal samples from Hainan gibbons inhabiting three groups and combined field surveys and DNA analysis to identify patterns of gene flow, genetic drift, inbreeding depression, and genetic variability in this Critically Endangered primate species. Based on the research of Bryant et al. (2016b), we selected 10 microsatellite markers to obtain detailed information on Hainan gibbon genetic diversity. Our goal was to use these data to develop an effective management strategy to protect the last remaining Hainan gibbon population.

## Materials and Methods

### Sampling and DNA Extraction

**FIGURE 1:**
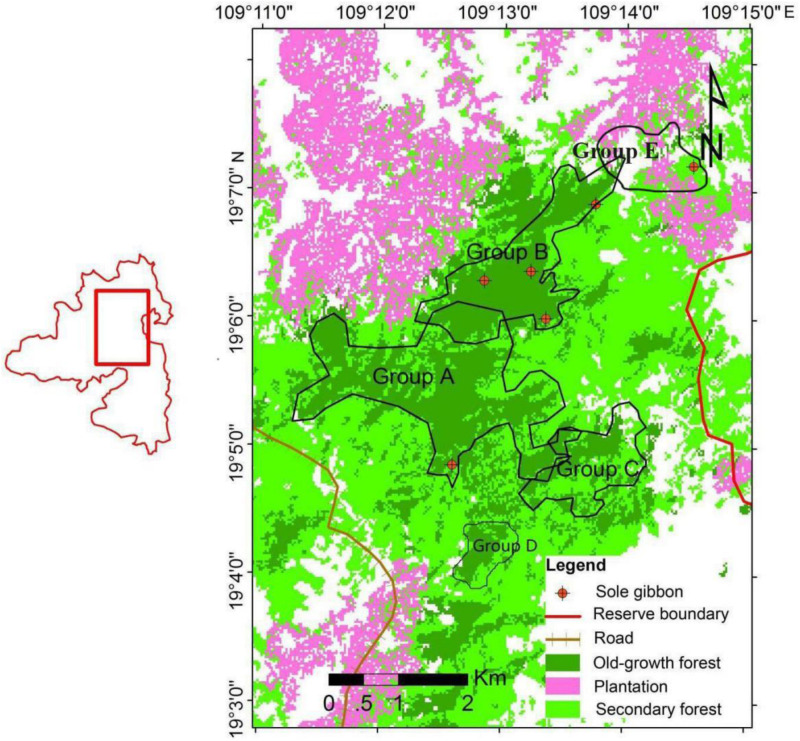
Distribution of the five remaining groups of Hainan gibbons (Group E was discovered in June 2020; the survey of this study does not include Group E).

We studied Hainan gibbons inhabiting the Bawangling National Nature Reserve (19N 02′–19N 08′, 109 E 02′–09 E 13′), Hainan, China (Figure 1). Male and female Hainan gibbons display each morning by producing a set of highly stereotypic vocalizations. From September 2017 to December 2018, we monitored these vocalizations to identify group location (for groups A, B, C, and D; we did not monitor group E) and at these locations we collected fecal samples noninvasively and immediately after defecation (for groups A, B, and C). High-temperature sterilized tweezers and petri dishes were used to collect fecal samples, which were stored in liquid nitrogen, and then kept cold using dry ice to transport the samples back to our laboratory for cryogenic storage. We also collected blood samples from two yellow-cheeked gibbons (*Hylobates gabriellae*) housed at the Nanning Zoo, Guangxi, China, and used these for comparative analyses.

We extracted DNA from 100 to 150 mg of feces using a QIAamp Fast DNA Stool Mini Kit and from 1 ml of blood using a QIAamp Blood & Cell Culture DNA Kit, following the manufacturer’s instructions. The extracted DNA was subjected to 0.8% agarose gel electrophoresis, GreenView nucleic acid dye staining, and the estimated concentration and purity (260/280, 260/230 value) were recorded using a UV transilluminator. At the same time, we employed a Qubit 3.0 fluorescence quantifier to accurately quantify the concentration of DNA.

### Identification of Polymorphic Markers and Genotyping

#### Microsatellite Loci

We tested 10 microsatellite loci previously described to be polymorphic in Hainan gibbons (Bryant et al., 2016b). Using the method of searching for microsatellite loci in the whole genome (Hou et al., 2018), and based on the reference genome of the northern white-cheeked gibbon (*Hylobates leucogenys*), 20 microsatellite loci were screened. To guarantee high-amplification success, particularly for DNA sourced from noninvasive samples, we selected microsatellites with >20 repeats and a product length of 100–300 bp. From the library of microsatellites that met our requirements, we randomly selected loci comprising different motifs for further optimization.

DNA extracts from two blood samples obtained from captive yellow-cheeked gibbons were used to amplify the 30 loci (Supplementary File 1). Polymerase chain reaction (PCR) amplification was performed in a 10-μl reaction volume, containing 1 μl of template DNA, 1 μl of forward and reverse primers, respectively, 5 μl of QIAGEN Multiplex PCR Master Mix (Qiagen), and 2 μg/μl of bovine serum albumin (BSA). The amplification conditions were 94°C for 15 min, 35 cycles at 94°C for 30 s, 43–65°C for 45 s, 72°C for 45 s, and final extension at 72°C for 5 min. Polymerase chain reaction products were separated on 3% agarose gels by electrophoresis to visually assess the amplification efficiency.

Next, we filtered the reliably sites from the *Hylobates gabriellae* blood sample amplifications and used the fecal samples of the Hainan gibbons to select appropriate microsatellite loci. Human blood was used for comparison to ensure that there was no contamination during the DNA extraction process.

PCR products were visualized on an ABI3730 XL Genetic Analyzer. Alleles were scored using GENEMARKER version 2.2.0. Readable peak: peak reading at ≤100 and lower intensity peak ≤50% of the higher intensity peak (the peak reads 50), otherwise the reading was abandoned and set to 0. According to genotyping criteria for the number of repeated experiments outlined by Taberlet and Luikart (1999), the length of allele fragments and the data were counted according to the integer multiples of 2, 3, and 4 bp in the length of different alleles. These data were transposed into an Excel table for subsequent population genetic testing.

#### Sex Markers

Three primers from Palle and Tina (2006), Valiere (2002), and Bolechova et al. (2016) were tested. We used known females and males to test the reliability of these primers for determining sex identification.

### Individual Identification

We used the MICROSATELLITE TOOLKIT to obtain individual genotype data from each fecal sample (Park, 2001; Zhan et al., 2007; Hou et al., 2018), and combined sex identification (see above) and field survey data to obtain individual identities.

### Data Assess

#### Genotyping

The presence of null alleles and scoring errors for each locus was estimated using MICRO-CHECKER v 2.2.3. Next, we used three indices to assess the reliability of genotyping. (1) Genotyping errors that resulted in allelic dropout (ADO), false alleles (FA), ADO, and FA were calculated using GIMLET v 1.1.3. (2). PCR success rates were estimated by calculating the percentage of successful PCRs that matched the consensus genotype (Wulstch et al. 2014). (3) For a specific locus i, we calculated the mean quality index (QI) of n samples for that locus using the following equation (Miquel et al., 2006).

(1) $${\text{QI}}_{i,}=\sum_{j=1}^{n}{\text{QI}}_{i,j}$$

Where *n* is the number of samples and QI*_*i*_*, *j* the quality index of the *i*th locus for the jth sample. QI*_*i*_*,*_*j*_* is estimated by the proportion of correct genotypes in three PCR replicates.

(2) $${\text{QI}}_{,j}=\sum_{i=1}^{m}{\text{QI}}_{i,j}$$

(3) $$Q\,\text{I}=\frac{\sum_{i=1}^{m}\sum_{j=1}^{n}Q\,{\text{I}}_{i,j}}{n\times m}$$

We further calculated the quality index of each sample using Eq. 2 (e.g., *j*th sample) and we calculated the global quality index using Eq. 3: In Eq. 3, *n* and *m* represent the numbers of loci and samples, respectively.

#### Polymorphic Information Content and Hardy–Weinberg Equilibrium

To investigate the suitability of our markers, we calculated the polymorphic information content (PIC), which is an estimate of the discriminating power of markers (ranging from 0 to 1, from no allelic variation to only new alleles) (Botstein et al., 1980). We also tested markers for deviation from Hardy–Weinberg equilibrium (HWE). We assumed that deviation from HWE would indicate genotyping problems, such as segregating null alleles or incorrectly distinguished alleles.

#### Individual Recognition Ability

The individual identification ability of each loci can be determined by calculating the PID (probability of identity) value (Valiere, 2002; Zhan et al., 2007). We used the software Gimlet v1.1.3 to calculate the values of PID and PIDsib. Judgment criteria: PID < 0.001 and PIDsib < 0.01 (Waits et al., 2001; Hou et al., 2018). According to the curve produced by plotting the PID value of each locus, the closer the curve is to the *X* axis, the stronger the individual recognition ability.

### Assessing Genetic Variation and Inbreeding

For each of the selected markers, we computed standard population genetic parameters of genetic variation. First, we calculated the expected heterozygosity (He), the observed heterozygosity (Ho), allelic frequencies, and the effective number of alleles. If the observed heterozygosity is lower than expected, this indicates inbreeding, while a higher than expected heterozygosity suggests the admixture of two previously isolated populations (Hartl and Clark, 1997). Furthermore, we determined inbreeding coefficients (FIS), with negative values indicating an excess of heterozygosity (Hedrick, 2000). We conducted all calculations in GeneALEx V.6.51 except the Wright F statistic (FIS), which we computed using FSTAT (version 2.9.3.) (Goudet, 2001).

### Parentage Analysis

We used COLONY V.2.0.6.4 to conduct kinship analyses in order to infer the theoretical parental pair (Jones and Wang, 2010). We used the criterion probability of ≤95% to determine kinship (parent–child, mother–child, parental pair) (Wang and Santure, 2009; Bryant et al., 2016b; Wang, 2017).

### Population Size Estimation

Based on linkage disequilibrium, we used NeEstimator V2.1 to calculate the effective population size.

### Allele Loss

To detect allelic loss in our study population, we used genotype data from the microsatellite loci and compared those results with the historical population of Hainan gibbons presented in Bryant et al. (2016b).

## Results

### Study Population

**FIGURE 2:**
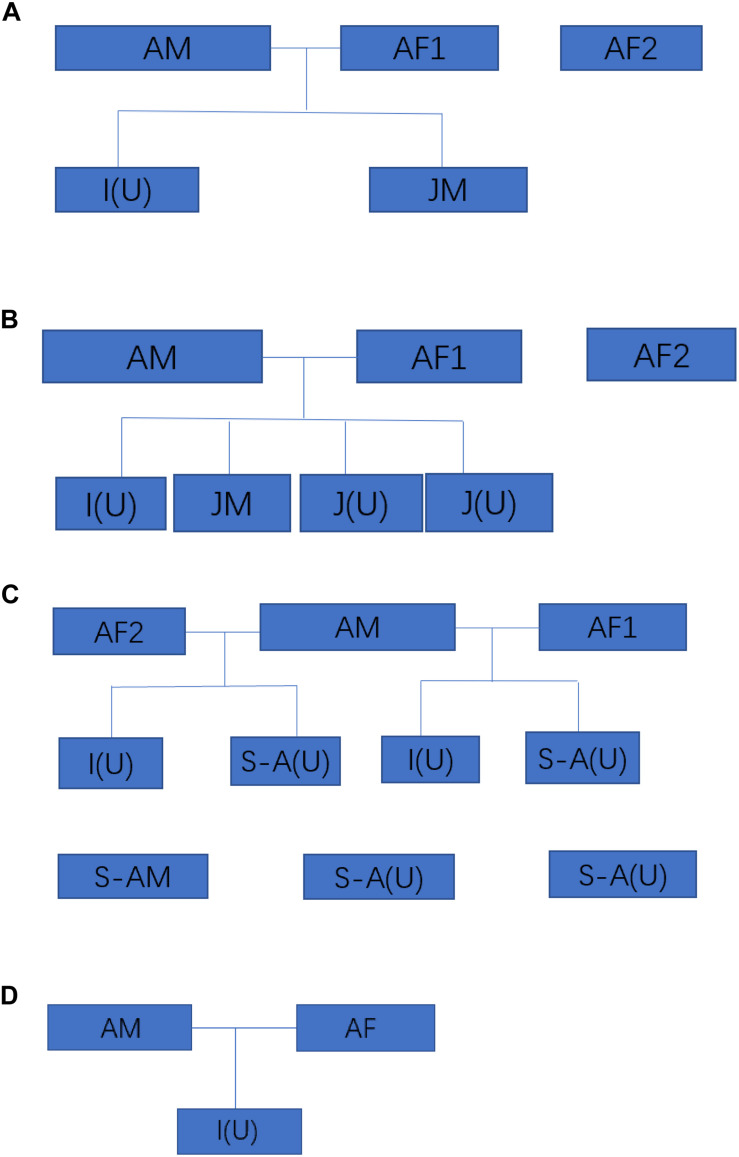
The size and composition four wild Hainan gibbon groups. AM represents an adult male, AF represents an adult female, S-A represents a subadult, JM represents a juvenile, I represents an infant, and (U) represents an adult, subadult, juvenile, or infant of unknown sex. Infants are 0–24 months of age, juveniles are >2–7 years of age, subadults are >7–9 years of age, and adults are >9 years of age.

We investigated the size and composition of four of the five remaining family groups of Hainan gibbons. The total population size of these four groups is 25 individuals (Figure 2). A total of 36 fecal samples were collected from three of these four groups, of which 6 samples were collected from Group A, 11 samples were collected from Group B, and 19 samples were collected from Group C. No genetic data were collected for Group D (Supplementary File 2).

### Characterization of Markers

#### Microsatellite Markers

Overall, 33% (10/30) of the microsatellite markers tested suitable for investigating genetic diversity in the Hainan gibbon population. These included 6 tetranucleotide and 4 dinucleotide loci with 4–9 alleles per locus (Table 1 and Supplementary File 3).

**TABLE 1:** Ten pairs of microsatellite loci and their primers.

**Locus**	**5′ modification**	**Duplication**	**Alleles**	**PCR product size/bp**
SSR15	HEX	AAT	3	232
SSR17	FAM	TATT	3	208
D2S367	FAM	CA	5	138–156
D5S1457	HEX	GATA	3	110–118
D7S817	TAM	GATA	6	130–148
D1S548	FAM	TATC	3	161–173
D5S1470	HEX	GATA	4	192–204
D6S265	TAM	CA	5	118–134
D20S206	TAM	GATA	2	132–144
DQcar	HEX	CT	4	86–104

#### Sex Markers

**FIGURE 3:**
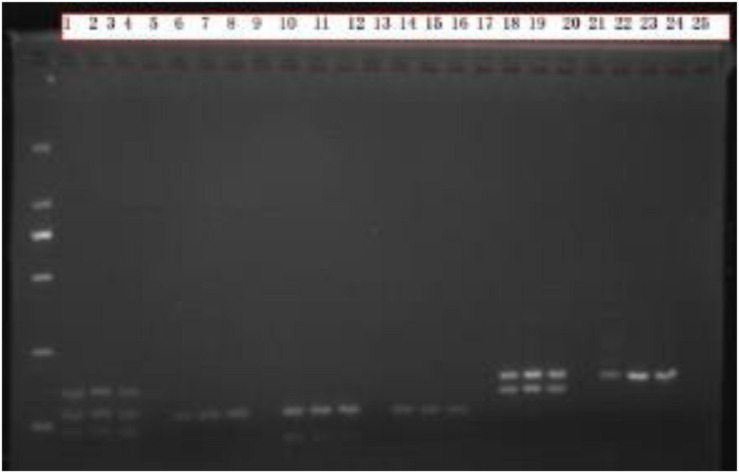
Sex identification electrophoresis test results. Males have 2–3 bands, females have only 1 band, and numbers 1–8 are the PCR products of Scheme A. The blank control group does not have any bands, the target band is bright, and the background is clean; Numbers 9–16 are the PCR products of Scheme B. The blank control group has no bands, the target bands have a clean background, and the bands are fuzzy; Numbers 17–25 are the PCR products of Scheme C. The blank control group has no bands, the target bands are bright, and the background is clean, but some samples have nonspecific amplification.

Comparing the electropherograms of the sex-marked PCR products, we found that PCR products numbered 1 to 8 had no bands in the blank control group, and the target bands were bright and the background was clean. Select A-UTXTUY_F1, A-UTXR1, A-UTYR1, A-SRY_F1, and A-SRY_R1 (Valiere, 2002) were found to be reliable as sex identification markers (Figure 3).

### Individual Identification

Combined with the results of sex identification, a total of 12 different individuals (4 females and 8 males) were identified. These 12 individuals included three residents of Group A (1 female and 2 males, accounting for 60% of this group), four residents of Group B (1 female and 3 males, accounting for 57% of this group), and five residents of Group C (2 females and 3 males, accounting for 50% of this group). Fecal samples of infants (wet and scattered after voided) could not be collected. Overall, the 12 genetically identified Hainan gibbons accounted for approximately 67% of the total number of individuals present in the A, B, and C Groups (Table 2).

**TABLE 2:** Individual identification of residents in the A, B, and C Hainan gibbon social groups.

**Group**	**No.**	**Sex**	**The same individual**	**Individual identification (combined with field investigation)**
A	A01	female	A03, A05	Female adult without infant
	A02	male		Male juvenile
	A04	male	A06	Male
B	B01	female	B01	Female adult with infant
	B02	male	B02	Male subadult “Anan”
	B06	male	B06	Male
	B07	male		Male juvenile
C	C07	male	C07	Male subadult
	C19	male	C19	Male subadult
	C06	male	C06	Male subadult
	C10	female	C10	Female adult with infant
	C08	female	C09, C11, C12, C16, C18	Female subadult

### Performance of the Microsatellite System in Hainan Gibbons

None of 10 loci showed allelic loss, null alleles, or reading errors caused by stuttering (shadow peak). The amplification success rate was 74.09–100%, and the average amplification success rate was 93.40%. The quality index was 0.766–1, and the average quality index was 0.891.

The PIC results ranged from 0.1948 to 0.6682, suggesting that our markers had high discriminating power. The 10 loci all conformed to a Harvin equilibrium, indicating that the selected 10 microsatellite loci were all neutrally inherited (Table 3).

**TABLE 3:** Number of alleles (Na), observed and expected heterozygosity (Ho, He), polymorphic information content (PIC), inbreeding coefficient (FIS), and Hardy–Weinberg deviation (P_*HWE*_) for 10 selected markers with the mean and standard deviation (SD) across all markers.

**Loci**	**Na**	**Ho**	**He**	**F*IS***	***P*_*HWE*_**	**PIC values**
SSR12	2	0.417	0.497	0.203	0.5939	0.3733
SSR17	3	0.750	0.517	–0.414	0.2839	0.4443
D1S548	3	0.750	0.642	–0.125	1.0000	0.5697
D5S1470	2	0.417	0.330	–0.222	1.0000	0.2755
D6S265	4	0.917	0.719	–0.235	0.9132	0.6682
D2S367	2	0.667	0.444	–0.467	0.2160	0.3457
D5S1457	2	0.333	0.278	–0.158	1.0000	0.2392
D7S817	2	0.750	0.469	–0.571	0.0922	0.3589
D20S206	2	0.250	0.219	–0.100	1.0000	0.1948
DQcar	2	0.833	0.486	–0.692	0.0631	0.368
Mean	2.400	0.608	0.460	–0.283		0.3838
S.D.	0.221	0.074	0.049	*n*/*a*		−

**FIGURE 4:**
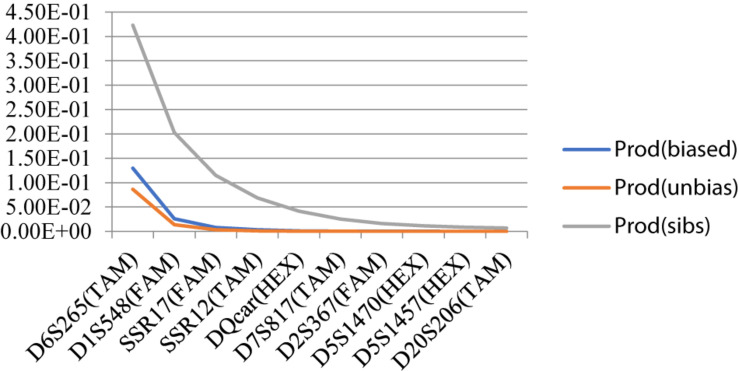
Individual identification system ability curve.

Individual Recognition Ability P(ID)DQcar was <0.001, indicating that only five sites were useful in recognizing two unrelated individuals. Similarly, P(ID)sib D5S1457 was <0.01, and thus using data from 9 sites allowed us to recognize two related individuals (Figure 4). Therefore, the individual identification system of 10 sites in this study was sufficient for individual recognition of Hainan Gibbon.

### Sex Ratio

We found that across all three of our main study groups, there was a greater number of male Hainan gibbons than female Hainan gibbons. Of the 12 genetically identified individuals, 8 were males and four were females. In addition, of the 7 individuals for which parentage was determined, 6 were males and one was female. Based on the results of individual DNA identification, although this population contains many more males than females, the observed sex ratios of the offspring in Groups A, B, and C deviate from an expected 1:1 (Table 4).

**TABLE 4:** Sex ratio of the Hainan gibbon study population.

**Sample**	**Female**	**Male**	**Sex ratio**	**Yates-corrected chi-square test against 1:1 *P* < 0.1**
All	4	8	1:2	χ^2^ = 1.333, *P* = 0.248
Group A	1	2	1:2	χ^2^ = 0.333, *P* = 0.564
Group B	1	3	1:3	χ^2^ = 1, *P* = 0.317
Group C	2	3	2:3	χ^2^ = 2, *P* = 0.655
All offspring	1	6	1:6	χ^2^ = 3.571, *P* = 0.059

### Genetic Variation and Inbreeding

The observed heterozygosity (Ho) ranged from 0.250 to 0.917 and expected heterozygosity (He) from 0.219 to 0.719 (Table 3). The mean observed heterozygosity (mean ± SD = 0.608 ± 0.074) was greater than the mean expected heterozygosity (mean ± SD = 0.460 ± 0.049) (Table 3), In other words, although we expected approximately 46% of individuals to be heterozygous at a given locus under random mating conditions, on average 60% of individuals were heterozygous. Similarly, the mean FIS was −0.283 (mean), with FIS consistently <0 for all 12 polymorphic loci, indicating an excess of observed heterozygosity (see Hedrick, 2000, for comparison). Thus, individuals in our three study groups were less closely related than expected under random mating.

### Parentage Analysis

**FIGURE 5:**
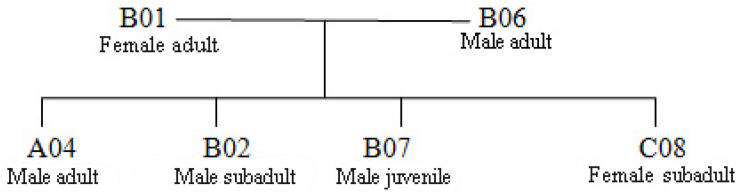
Genetic maps of 3 Hainan gibbon groups.

We constructed a genetic pedigree (Figure 5) based on the results of kinship analysis (Table 5), Six individuals in Groups A, B, and C were identified as parent–child or full-sibs.

**TABLE 5:** Parent–offspring pair inferred using the software COLONY.

**Offspring ID**	**Inferred father**	**Inferred mother**	**Probability**
A04	B06	B01	1.000
B02	B06	B01	0.9744
B07	B06	B01	0.9892
C08	B06	B01	0.9945

*The letters A, B, and C refer to the groups in which these individuals currently reside.*

### Population Size

The effective population size of the Hainan Gibbon is 5 (Table 6). The actual population is 33 individuals, and the ratio of effective population to actual population is 1:6.

**TABLE 6:** Effective population size estimates of Hainan gibbon.

	**Estimated Ne (*P*_*crit*_ = 0.010)**	
	Ne	95% confidence interval
Hainan gibbon	5.3	5.3

### Allele Loss

**FIGURE 6:**
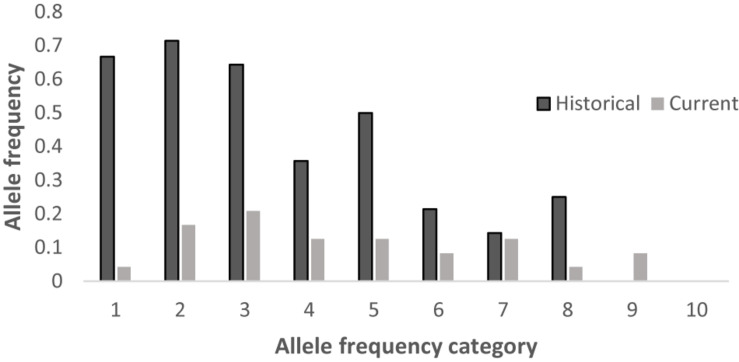
Distribution of allele frequencies for the historical and current populations across 8 loci.

We compared the published microsatellite genotyping data of the historical population of Hainan gibbons with our results (Bryant et al., 2016b). Among the 8 microsatellite loci we examined, the number of alleles at 7 microsatellite loci in the present Hainan gibbon population has decreased, allelic loss has occurred, and allelic frequency has changed (Supplementary File 4 and Table 7). Alleles fall into frequency classes (1–10). The current population showed fewer alleles in low-frequency classes and more in higher-frequency classes. This suggests that some alleles with low frequency recently have been lost in this population (Figure 6). This is consistent with the profile of a population characterized by a marked decline in genetic diversity.

**TABLE 7:** The difference in the number of alleles at 8 microsatellite loci between the existing population and the historical population.

	**Current population**	**Historical population**

**Locus**	**Number of alleles**	**Number of alleles**
D1S548	3	3
D5S1470	2	3
D6S265	4	5
D2S367	2	4
D5S1457	2	3
D7S817	2	5
D20S206	2	1
DQcar	2	4

## Discussion

### Sample Collection and Suitability of Selected Markers

This study represents the first integrated investigation of the population demography and genetic diversity of the critically endangered Hainan gibbon. We have conducted long-term field observations of this species, and in the present study we also collected and analyzed DNA (from feces) from 50 to 60% of all residents in three of the five remaining wild groups. Our DNA sampling coverage was greater than in any previous study of this species (Li et al., 2010; Bryant et al., 2016b). In this regard, as pointed out by Tajima (1982), a DNA sequencing sample size of >10 is expected to result in limited variance and a representative genetic profile.

Prior to 2007, field surveys reported the existence of only two wild groups of Hainan gibbons, Group A and Group B (Zhou et al., 2008). Subsequently, Group C was discovered in 2011. Group C was formed by the reorganization of individuals from groups A and B (Deng et al., 2015). In 2015, Bryant et al. (2016a) used the method of acoustic playback to identify a newly formed group of Hainan gibbons (Group D). In 2020, we located a fifth group of Hainan gibbons (Group E). Although our study did not collect genetic samples from residents of Group D or Group E, we assume that these groups were founded by members of Groups A, B, and/or C, and therefore from the perspective of population structure, the samples we collected are likely to reflect the genetic information present in Hainan gibbons.

The microsatellite markers used in this study fulfill important genetic and technical criteria that allow for high levels of precision and efficacy using high-throughput genotyping, which greatly improves the reliability of the genetic information (Butler et al., 2001). Twenty microsatellite loci were screened from the reference genome of *Hylobates leucogenys*, a close relative of the Hainan gibbon. The availability of several reference genomes provides valuable resources for genetic marker identification, and this resulted in simpler procedures for sample preparation and provided a larger amount of genetic information than other types of sequencing data (e.g., transcriptome). Compared with other types of genetic markers (SNPs), microsatellites have relatively high amplification success rates, especially for noninvasive samples, and have been used widely for individual identification in avian populations (Hou et al., 2018). Our results show that the 10 microsatellites used in this study provided reliable information on the sex and genotype of individual Hainan gibbons, provided high confidence paternity assignment, presented a relatively high level of polymorphic information and genetic variation, and resulted in a high accuracy of allelic characterization and a low occurrence or absence of mutations.

### Declining Genetic Diversity

At the microsatellite level, the genetic diversity of the Hainan gibbon population is significantly lower than that reported in other gibbon species. For example, the number of alleles (Na) of the lar gibbon (*Hylobates lar*) is 7 and the expected heterozygosity (He) is 0.725 (Chambers et al., 2004); the number of alleles (Na) in Müller’s gibbon (*Hylobates muelleri*) is 14.8 (Oka and Takenaka, 2001). Similarly, the Endangered Borneo elephant (*Elephas maximus*), which today is only distributed in the northeastern part of Sabah, Malaysia, and has a remaining population of only 2,000 individuals, also is characterized by low genetic diversity (Ho = 0.14–0.41) and significant inbreeding (Fis = 0.14–0.38) (Benoit et al., 2016). In the case of the Mexican howler monkey (*Alouatta palliata mexicana*), a Critically Endangered subspecies endemic to Mexico, extensive habitat loss and fragmentation over the past 30 years have resulted in a major population decline. Presently, this primate taxon is distributed in four forest fragments in the state of Veracruz, Mexico. Genetic testing revealed that haplotype diversity and nucleotide diversity (*h* = 0.486; π = 0.0007) are extremely low compared with other Neotropical primates (Jacob et al., 2014).

The low genetic diversity of the remaining Hainan gibbon population is consistent with their severe population decline (99.4%), which has occurred over the past 70 years (Zhou et al., 2008). Their current population size of only 33 individuals is the result of extreme deforestation and forest fragmentation that has decreased their remaining area of suitable habitat from 27,784 km^2^ (Zhou et al., 2005) to approximately 16 km^2^ (Zhou et al., 2008). The number of alleles in the existing population, and therefore its genetic diversity, is far less than historically reported (Bryant et al., 2016b) and extremely low compared to other primate populations.

### Probability of Inbreeding in the Future

**FIGURE 7:**
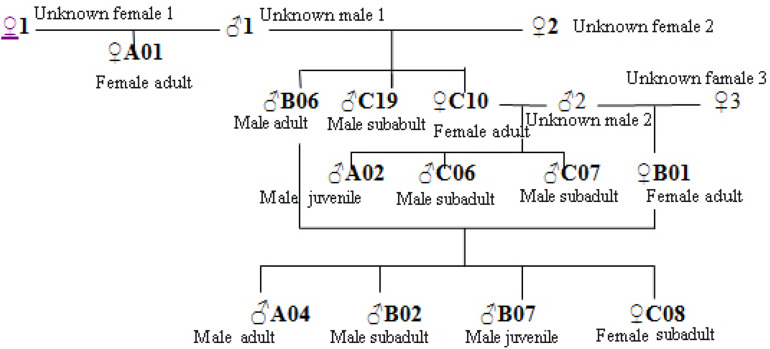
Kinship diagram of individuals in Groups A, B, and C. Based on these results, both males and females transfer between established groups, and given the existence of new groups (D and E), individuals periodically form new groups.

The social and breeding system of Hainan gibbons is described as monogamous (Liu et al., 1989; Zhou et al., 2008). When the number of reproducing males in a population is small, binomial sampling error occurs, and the frequency of alleles contributed by paternal and maternal lines is unequal (Storz et al., 2001). Our analysis indicated no evidence of inbreeding in the existing population of Hainan gibbons. The excessive heterozygotes (FIS <0) reveal that mating in the current population is biased toward individuals with relatively distant relationships. Combining kinship analysis with behavioral field observation, the genetic pedigrees of the 12 identified Hainan gibbons indicate high levels of relatedness among individuals in Groups A, B, and C (Figure 7). Also, the offspring sex ratio deviates significantly from 1:1; given the large number of males in the population, we anticipate high levels of inbreeding in the future. Inbreeding may build up over a much longer time frame than relatedness. Next, we will use genome technology to deeply analyze the mechanism of inbreeding.

### Long-Term Survival Goals

The remaining area of suitable habitat for Hainan Gibbons is extremely small and highly fragmented. Low genetic diversity of the population is likely the result of founder effect and a sharp decline in effective population size (Frankham, 1997; Groombridge et al., 2009). Compared with the effective population size of the historical population (Ne = 1162.96), the effective population size of the existing population (Ne=5.3) has been significantly reduced. When the effective population size (Ne) is <50, population viability decreases significantly (Madsen et al., 1999). The long-term evolutionary survival of any population is expected to require an Ne ≥1000 (Frankham et al., 2014).

In the 1970s, the total Hainan gibbon population in the Bawangling National Nature Reserve was 7–8 individuals (Zhou et al., 2005). It has taken more than 40 years for this population to reach its current size of 33 individuals (rate of 0.625 individuals added to the population per year). Given their relatively slow life history (individuals do not reach adulthood until >9 years of age and females give birth every 2–4 years), the long-term viability of the current Hainan gibbon population remains doubtful unless there is an immediate and significant reforestation effort and targeted programs to protect the remaining Hainan gibbon population.

### Conservation and Management

Given their limited remaining habitat, low genetic diversity, extremely small effective population size, and high potential inbreeding for inbreeding, we plan to establish a genetic profile for each of the remaining 33 Hainan gibbons. This will allow us to monitor both behaviorally and genetically the degree of population inbreeding and potential for disease transmission and, if necessary, intervene to promote increased genetic variability and population health. We also propose that the Bawangling National Nature Reserve immediately initiates a program to professionally train reserve staff so that they can continuously monitor the behavior, diet, and demography of individuals in all five gibbon groups and reforest and restore the current fragmented habitat in ways to minimize the negative impact of human disturbance on gibbon survivorship. Finally, efforts must be made to strengthen communication and cooperation among all stakeholders and reach a scientifically based consensus on measures that must be taken to ensure the long-term survival of the last remaining Hainan gibbon population.

## Data Availability Statement

The datasets presented in this study can be found in online repositories. The names of the repository/repositories and accession number(s) can be found in the article/Supplementary Material.

## Ethics Statement

Ethical review and approval was not required for the animal study because Noninvasive sampling method.

## Author Contributions

JZ, JC, and NX designed the study. YG wrote the manuscript and analyzed the data. LH completed the experiments and analyzed the data. PG wrote the manuscript. TL and GL conducted the field investigations. All authors contributed to the article and approved the submitted version.

## Conflict of Interest

The authors declare that the research was conducted in the absence of any commercial or financial relationships that could be construed as a potential conflict of interest.
