# Supplementary material for: The Genetic Status of the Critically Endangered Hainan Gibbon (Nomascus hainanus): A Species Moving Toward Extinction
Source: Front Genet. 2020 Dec 4;11:608633. doi: 10.3389/fgene.2020.608633 (PMC7746834; doi:10.3389/fgene.2020.608633)
Supplement: Supplementary file 1 [file Data_Sheet_1.docx]

Supplementary File 1. Information detailing the selected 30 microsatellite loci primers

| **Loci ID** | **Product size/bp** | **Motif** | **Left primer** | **Right primer** |
| --- | --- | --- | --- | --- |
| D1S548 | 161-173 | TATC | GAACTCATTGGCAAAAGGAA | GCCTCTTTGTTGCAGTGATT |
| D2S367 | 138-156 | CA | TTCTTTGGTCTAAGGGTCAC | AGCTTCTTGTTCACAGGTGT |
| D5S1457 | 110-118 | GATA | TAGGTTCTGGGCATGTCTGT | TGCTTGGCACACTTCAGG |
| D5S1470 | 192-204 | GATA | CATGCACAGTGTGTTTACTGG | TAGGATTTTACTATATTCCCCAGG |
| D6S265 | 118-134 | CA | ACG TTC GTA CCC ATT AAC CT | ATCGAGGTAAACAGCAGAAA |
| D7S817 | 130-148 | GATA | TTGGGACCTCTTATTTTCCA | GGGTTCTGCAGAGAAACAGA |
| D9S302 | 188-192 | Tetra | GGGGACAGACTCCAGATACC | GCGACAGAGTGAAACCTTGT |
| D17S804 | 145-161 | Di | CCTGTGCTGCTGATAACC | CATTGTGATGAG ATGTCAT |
| D20S206 | 132-144 | GATA | TCCATTATTCCCCTCAAACA | GGTTTGCCATTCAGTTGAGA |
| DQcar | 86-104 | CT | GAAACATATATTAACAGAGACAGACAAA | CATTTCTCTTCCTTATCACTTCATA |
| NL-SSR01 | 260 | CTTC | GCAACAAAATCCTCCCTCCC | TGGAACTGAGAGTCCATTAAACC |
| NL-SSR02 | 156 | TAT | AAGTAGGTGGCGGATTCAGT | GTTGACAGGCTAACGGGTG |
| NL-SSR03 | 205 | GT | TGACCATGGCCTAATCAGCT | CTTTCCCTTTCTGCTCCCCT |
| NL-SSR04 | 160 | AAT | GAGCTGAGATTGTGCCACTG | TGTCCTCCGTCCGACTAATG |
| NL-SSR05 | 214 | AAC | AATCCCAGCTACTCAGGAGG | GCCTTCAGTCTGGACCTCAT |
| NL-SSR06 | 181 | TCA | AGGAAACAGAGGCTCCAGTG | TAGTGGCCAAGATACGACCC |
| NL-SSR07 | 213 | ATT | GGTTCCTGCCCTGATCTCAA | AATCGCTTGAACCTGGGAGA |
| NL-SSR08 | 204 | ATT | GTGGTGCATGCCTGTAGTTC | TGCTGCTCACCCTTACTTCA |
| NL-SSR09 | 173 | CTA | GGTTGTGCTGTAGATGACTTCC | GAGTTAAGGTACCTGCACAGC |
| NL-SSR10 | 214 | AAAG | GAGCCAAGATCACGCCATTG | CCTTCCAGGTGATTCTAATGCA |
| NL-SSR11 | 201 | ATCT | CCTTTGACATGGAACCCTAGC | GGTGACAGAGTGAGACCCTG |
| NL-SSR12 | 194 | AC | CACCGATGTTCATAGCAGCA | GCTCATCCATGTTGTAGCAGA |
| NL-SSR13 | 220 | GT | CCTCCCAACAAGCTGAGACT | AGGTGCCTGTAATCCCAACT |
| NL-SSR14 | 243 | TAT | AGTCCCTGGGCTCAAATGAT | ATGCCTGCAGTCCTAGCTAC |
| NL-SSR15 | 232 | AAT | CAGATGTGGGGCTAGAGGAG | CACCCAGTGTTTGCCTTGAG |
| NL-SSR16 | 177 | AAGA | TCGCACCATTACATTCCAGC | GTCATTCAGGAGCATGTTGTTT |
| NL-SSR17 | 208 | TATT | TGGCAAGCAATACGATCAACA | CGCTTGAACCAGTGAGTCAG |
| NL-SSR18 | 205 | TTAT | TGAACTTGAGTCCTGGAGCC | GGAGGTCGAGACTGCAATGA |
| NL-SSR19 | 165 | GATA | AGGAGGCAGATGATTATGATGAC | ACAGCCTTACAGAGACAGCT |
| NL-SSR20 | 240 | TATC | GGGACCCTGTGATCATGTGA | AAGCAGCCAACTCCAGAAAC |

Supplementary File 2 Fecal sample collection information

| Group A | | | | |
| --- | --- | --- | --- | --- |
| Samples | Date | Local | Individual information | GPS |
| 1 | 2017.05.18 | Daankou | Adult female | N：19°05′55.95″  E：109°14′01.57″  H：955 |
| 2 | 2017.06.27 | Honghegu | Juvenile male | N：19°06′00.58″  E：109°13′46.60″  H：984 |
| 3 | 2017.06.27 | Honghegu | unknown | N：19°05′59.83″  E：109°13′49.14″  H：1007 |
| 4 | 2017.06.27 | Honghegu | Adult male | N：19°05′48.47″  E：109°13′34.90″  H：979 |
| 5 | 2017.06.27 | Honghegu | unknown | N：19°05′49.01″  E：109°13′34.24″  H：1002 |
| Group B | | | | |
| Samples | Date | Local | Individual information | GPS |
| 1 | 2017.06.03 | Hengganggou | Adult female | N：19°07′11.18″  E：109°13′07.34″  H：939 |
| 2 | 2017.06.03 | Hengganggou | Adult male | N：19°07′11.18″  E：109°13′07.34″  H：939 |
| 3 | 2017.06.04 | Nanbangou | Subadult | N：19°07′01.39″  E：109°13′22.38″  H：1084 |
| 4 | 2017.06.04 | Nanbangou | unknown | N：19°06′59.37″  E：109°13′24.84″  H：1038 |
| 5 | 2017.06.04 | Nanbangou | Subadult | N：19°06′58.95″  E：109°13′25.03″  H：1034 |
| 6 | 2017.06.05 | Nanbangou | Adult male | N：19°07′01.19″  E：109°13′33.50″  H：1058 |
| 7 | 2017.06.05 | Nanbangou | Juvenile male | N：19°07′01.18″  E：109°13′33.83″  H：1046 |
| 8 | 2017.06.05 | Nanbangou | Adult female | N：19°07′00.73″  E：109°13′34.58″  H：1069 |
| 9 | 2017.06.05 | Nanbangou | Juvenile male | N：19°07′01.55″  E：109°13′34.16″  H：1035 |
| 10 | 2017.06.05 | Nanbangou | unknown | N：19°07′00.81″  E：109°13′34.18″  H：1047 |
| 11 | 2017.06.05 | Nanbangou | unknown | N：19°07′00.98″  E：109°13′34.19″  H：1054 |
| Group C | | | | |
| Samples | Date | Local | Individual information | GPS |
| 1 | 2017.02.18 | Miaocun | Subadult | N：19°05′53.09″  E：109°14′26.71″  H：944 |
| 2 | 2017.02.18 | Miaocun | Adult male | N：19°05′53.09″  E：109°14′26.71″  H：944 |
| 3 | 2017.02.18 | Miaocun | Adult female | N：19°05′53.09″  E：109°14′26.71″  H：944 |
| 4 | 2017.02.28 | Miaocun | unknown | N：19°05′35.21″  E：109°14′40.58″  H：842 |
| 5 | 2017.03.03 | Miaocun | Subadult | N：19°05′26.68″  E：109°15′01.39″  H：730 |
| 6 | 2017.03.03 | Miaocun | Sub-adult | N：19°05′26.68″  E：109°15′01.39″  H：730 |
| 7 | 2017.03.03 | Miaocun | Sub-adult | N：19°05′27.16″  E：109°15′01.51″  H：713 |
| 8 | 2017.03.03 | Miaocun | Adult female | N：19°05′27.23″  E：109°15′01.39″  H：714 |
| 9 | 2017.03.03 | Miaocun | unknown | N：19°05′26.94″  E：109°15′01.37″  H：716 |
| 10 | 2017.03.03 | Miaocun | Adult female | N：19°05′26.73″  E：109°15′01.28″  H：721 |
| 11 | 2017.03.03 | Miaocun | unknown | N：19°05′27.38″  E：109°15′01.08″  H：715 |
| 12 | 2017.03.03 | Miaocun | unknown | N：19°05′27.56″  E：109°15′01.52″  H：708 |
| 13 | 2017.03.03 | Miaocun | unknown | N：19°05′27.69″  E：109°15′01.48″  H：706 |
| 14 | 2017.03.03 | Miaocun | unknown | N：19°05′27.69″  E：109°15′01.48″  H：706 |
| 15 | 2017.03.06 | Miaocun | unknown | N：19°05′41.20″  E：109°14′29.01″  H：833 |
| 16 | 2017.03.06 | Miaocun | unknown | N：19°05′40.81″  E：109°14′29.12″  H：833 |
| 17 | 2017.03.06 | Miaocun | unknown | N：19°05′43.57″  E：109°14′33.82″  H：910 |
| 18 | 2017.03.07 | Miaocun | unknown | N：19°05′40.70″  E：109°14′47.82″  H：813 |
| 19 | 2017.03.07 | Miaocun | Sub-adult | N：19°05′39.12″  E：109°14′47.85″  H：804 |

Supplementary File 3. Genotype data of 10 microsatellites in 12 Hainan gibbons successfully identified

| **No.** | **Sex** | **SSR12** | | **SSR17** | | **D1S548** | | **D5S1470** | | **D6S265** | | **D2S367** | | **D5S1457** | | **D7S817** | | **D20S206** | | **DQcar** | |
| --- | --- | --- | --- | --- | --- | --- | --- | --- | --- | --- | --- | --- | --- | --- | --- | --- | --- | --- | --- | --- | --- |
| A01 | Female | 186 | 192 | 216 | 220 | 163 | 167 | 198 | 202 | 131 | 139 | 144 | 144 | 108 | 112 | 181 | 185 | 136 | 136 | 106 | 106 |
| A02 | Male | 192 | 192 | 212 | 220 | 163 | 163 | 198 | 198 | 131 | 133 | 144 | 154 | 108 | 112 | 181 | 185 | 136 | 136 | 92 | 106 |
| A04 | Male | 186 | 186 | 212 | 220 | 167 | 175 | 198 | 198 | 123 | 133 | 144 | 154 | 108 | 108 | 181 | 185 | 136 | 148 | 92 | 106 |
| B01 | Female | 186 | 186 | 216 | 220 | 167 | 175 | 198 | 198 | 131 | 133 | 144 | 154 | 108 | 108 | 181 | 181 | 136 | 148 | 92 | 106 |
| B02 | Male | 186 | 186 | 220 | 220 | 167 | 167 | 198 | 202 | 123 | 133 | 144 | 154 | 108 | 108 | 181 | 181 | 136 | 136 | 92 | 106 |
| B06 | Male | 186 | 192 | 212 | 220 | 167 | 167 | 198 | 202 | 123 | 139 | 144 | 144 | 108 | 108 | 181 | 185 | 136 | 136 | 92 | 106 |
| B07 | Male | 186 | 192 | 212 | 220 | 167 | 175 | 198 | 202 | 123 | 131 | 144 | 154 | 108 | 108 | 181 | 185 | 136 | 136 | 92 | 106 |
| C06 | Male | 192 | 192 | 212 | 220 | 163 | 175 | 198 | 198 | 131 | 133 | 144 | 144 | 108 | 112 | 181 | 185 | 136 | 136 | 92 | 106 |
| C07 | Male | 192 | 192 | 212 | 220 | 163 | 175 | 198 | 198 | 133 | 133 | 144 | 154 | 108 | 108 | 181 | 181 | 136 | 148 | 92 | 106 |
| C08 | Female | 186 | 186 | 220 | 220 | 167 | 175 | 198 | 198 | 131 | 139 | 144 | 154 | 108 | 108 | 181 | 185 | 136 | 136 | 106 | 106 |
| C10 | Female | 186 | 192 | 212 | 220 | 163 | 167 | 198 | 198 | 131 | 133 | 144 | 144 | 108 | 112 | 181 | 185 | 136 | 136 | 92 | 106 |
| C19 | Male | 186 | 192 | 220 | 220 | 163 | 167 | 198 | 202 | 133 | 139 | 144 | 154 | 108 | 108 | 181 | 185 | 136 | 136 | 92 | 106 |
| Control group | - | 168 | 172 | 190 | 190 | 163 | 163 | 194 | 202 | 125 | 129 | 156 | 158 | 112 | 116 | 170 | 174 | 164 | 168 | 98 | 100 |

Supplementary File 4. Data on the number and frequency of alleles at 8 microsatellite positions in the existing population of Hainan gibbons and the historical population provided by Bryant et al. (2016a).

|  | **Current population** | | **Historical population** | |
| --- | --- | --- | --- | --- |
| **Locus** | **Allele** | **Allele frequency** | **Allele** | **Allele frequency** |
| D1S548 | 163 | 0.292 | 161 | 0.357 |
|  | 167 | 0.458 | 165 | 0.429 |
|  | 175 | 0.250 | 173 | 0.214 |
| D5S1470 | 198 | 0.792 | 192 | 0.250 |
|  | 202 | 0.208 | 196 | 0.667 |
|  |  |  | 204 | 0.083 |
| D6S265 | 123 | 0.167 | 118 | 0.500 |
|  | 131 | 0.292 | 126 | 0.214 |
|  | 133 | 0.375 | 128 | 0.071 |
|  | 139 | 0.167 | 132 | 0.071 |
|  |  |  | 134 | 0.143 |
| D2S367 | 144 | 0.667 | 138 | 0.357 |
|  | 154 | 0.333 | 142 | 0.357 |
|  |  |  | 152 | 0.214 |
|  |  |  | 156 | 0.071 |
| D5S1457 | 108 | 0.833 | 110 | 0.714 |
|  | 112 | 0.167 | 114 | 0.214 |
|  |  |  | 118 | 0.071 |
| D7S817 | 181 | 0.625 | 130 | 0.429 |
|  | 185 | 0.375 | 136 | 0.214 |
|  |  |  | 140 | 0.214 |
|  |  |  | 144 | 0.071 |
|  |  |  | 148 | 0.071 |
| D20S206 | 136 | 0.875 | 132 | 1.000 |
|  | 148 | 0.125 |  |  |
| DQcar | 92 | 0.417 | 86 | 0.071 |
|  | 106 | 0.583 | 90 | 0.143 |
|  |  |  | 102 | 0.643 |
|  |  |  | 104 | 0.143 |
